# Aging and oral squamous cell carcinoma development: the role of cellular senescence

**DOI:** 10.3389/froh.2023.1285276

**Published:** 2023-10-12

**Authors:** Sven Eric Niklander, Pablo Aránguiz, Fernando Faunes, René Martínez-Flores

**Affiliations:** ^1^ Unit of Oral Pathology and Oral Medicine, Faculty of Dentistry, Universidad Andres Bello, Viña del Mar, Chile; ^2^ Escuela de Química y Farmacia, Facultad de Medicina, Universidad Andres Bello, Viña del Mar, Chile; ^3^ Departamento de Ciencias Biológicas, Facultad de Ciencias de la Vida, Universidad Andres Bello, Viña del Mar, Chile

**Keywords:** cellular senescence, oral cancer, mouth neoplasm, oral potentially malignant disorder, SASP, senotherapeutics

## Abstract

The gradual accumulation and inadequate renewal of senescent cells over time drive organismal aging. Senescent cells undergo altered gene expression and release inflammatory mediators collectively termed the senescence-associated secretory phenotype (SASP), which significantly contributes to a spectrum of age-related disorders, including cancer. In the context of carcinogenesis, the SASP produced by senescent cells has been implicated in the promotion of epithelial cancers, including oral squamous cell carcinoma (OSCC), the most common form of oral cancer. Senescent cells within the tumor microenvironment release factors that amplify the growth and invasiveness of neighboring cancer cells. Senotherapeutics, including senolytics and senomorphics, emerge as promising modalities to target senescent cells and their associated inflammatory factors, thereby opening novel avenues for augmenting the efficacy of cancer treatments. Here, we review the general aspects of cellular senescence, focusing on the relation between senescence-related inflammation with cancer development. We also analyze the available evidence linking cellular senescence with OSCC, highlighting possible clinical applications.

## 1. Introduction

The relation between organism aging and the development of different diseases (age-related diseases) is not new and is well accepted. Organism aging happens due to the accumulation and lack of renewal of aged cells across time (1). Cell aging or cellular senescence is a cellular state in which the growth capabilities of cells are irreversible lost in response to different stressors (2). An important feature of senescent cells is that they change their gene expression profile and develop an inflammatory secretome known as the senescence-associated secretory phenotype (SASP) (3, 4). The accumulation of senescent cells therefore can be detrimental, as the inflammatory factors secreted as part of the SASP can act on neighbor cells either predisposing, triggering or promoting the development of different diseases (5–7).

Many age-related diseases are influenced by the accumulation of senescent cells. This led to the development of new types of drugs, known as senotherapeutics. Senotherapeutics are drugs aimed to specifically target and eliminate senescent cells (senolytics) or to decrease the abundancy of inflammatory factors present in the SASP (senomorphics) (8). Although this field is relatively new, there are already human clinical trials reporting some of these drugs to be beneficial for the treatment of idiopathic pulmonary fibrosis (9) and many more are currently being held (https://clinicaltrials.gov/search?cond=cancer&intr=navitoclax).

There is robust evidence supporting stromal senescent cells as promoter of epithelial cancers of different origins. Recent studies have also suggested senescent cells to play a role in oral cancer squamous cell carcinoma (OSCC) development (10), progression (11) and therapy resistance (12). This could have an impact on the way we currently treat oral-precancerous lesions and OSCC. Here, we review the general aspects of cellular senescence, focusing on the relation between senescence-related inflammation with cancer development. We also analyze the available evidence linking cellular senescence with OSCC, highlighting possible clinical applications.

## 2. Cellular senescence

Senescence can be induced by different stimuli, including replicative stress, oxidative stress, oncogene signaling and DNA damage (13). Independent of the inductor, senescent cells acquire morphological alterations that differentiate them from non-senescent cells. There are 5 hallmarks of cellular senescence representing structural, epigenetic and signaling alterations: (i) chromatin reorganization, (ii) cell cycle arrest, (iii) metabolic adaptation, (iv) modifications of the lysosomal compartment and v) development of a secretory phenotype (SASP) (14, 15). There is no universal marker to identify senescent cells. The most used markers are the activity of senescent-associated β-galactosidase (SA-β-GAL), the expression of LaminB1, p16 and p21, the identification of senescence-associated heterochromatin foci (SAHFs) and the accumulation of DNA damage response (DDR) proteins, such as phosphorylated (γ) H2A. However, many others have been reported including the lack of expression of ki67 and the increased secretion of IL-6 and IL-8 SASP factors (15). None of the aforementioned features can be used by themselves to identify senescent cells, and a combination of two to four of these markers should be used (16).

Depending on the context, senescence can result in both beneficial and detrimental effects. In young individuals’ senescence contributes to tumor suppression and wound healing, primary by stopping the cell cycle and by secreting specific factors as part of the SASP (17–20). However, in older individuals or upon consistent and chronic damage, senescent cells accumulate in tissues. This contributes to tissue dysfunction, chronic inflammation, and age-related disorders, including cardiovascular diseases, fibrosis, diabetes, neurological disorders, and paradoxically cancer (21–23). These negatives effects are also attributed to the SASP. Due to these antagonistic effects, senescence is considered a double-edge sword in health and disease (24).

## 3. Cellular senescence as epithelial cancer promoter

Whether a senescent cell that has irreversible exited the cell cycle can re-enter and become malignant is still under study (25, 26). Reversing senescence is not a common feature and is not the mechanism by which cellular senescence is considered to have pro-tumorigenic effects. The pro-tumorigenic effects of senescence are explained by the SASP. Neighbor non-cancerous senescent cells (or cancerous cells induced to senesce because of radiation or chemotherapy) secrete soluble factors that can be used by pre-malignant or malignant cells for their advantage, which is changing the current understanding of cancer biology. In fact, senescent cells have been recently proposed as an emerging hallmark of cancer with enabling/promoting capabilities (27).

### 3.1. The SASP: regulation and composition

Different experimental models have suggested that the composition of the SASP varies between cell types and the senescence inducer (28). Nevertheless, a recent report which compared the SASP after inducing senescence under two different modalities in 13 different cancer cell types found the expression of the SASP to be more influenced by the cell type rather than the senescence inductor (4). Although the composition of SASP is widely heterogenous and more than 50 different soluble factors might be overexpressed (3), there is substantial overlap among SASPs, with specific proteins being almost invariably, including IL-1α, IL-6, IL-8, MMP-1, MMP-3, MMP-10 (29).

Virtually every cell that senesce will develop a SASP, although there are reports from animal studies in which under specific conditions cell senesced without developing a secretory phenotype (19). Diverse signaling pathways are associated to induce and maintain the SASP, including: phosphoinositide-3-kinase (PI3K) (30), mammalian target of rapamycin (mTOR) (31–33), p38MAPK (34), STAT3 (35), GATA4 (36), cGAS/STING (37–39), IL-1 signaling pathway (40) and Rho-kinase (41). These pathways have one thing in common, to activate NF-kB and/or CEBPβ transcription factors as downstream effectors (42).

### 3.2. Senescence and carcinomas

Classically, the accumulation of genetic and epigenetic changes in target cells has been considered as the primary cause of carcinoma development (43). However, that simplistic view has changed, based on the finding that pre-cancerous epithelial lesions with “cancer-associate mutations” may never progress into cancer (44) with substantial evidence suggesting that the final trigger for developing the malignant phenotype could be a micro-environmental change (45, 46). The current knowledge supports the idea that solid tumors, such as carcinomas, are not just clonally evolved epithelial cells that have accumulated a critical number of cancer-predisposing mutations, but rather dysfunctional tissues where the mesenchymal component (stroma) plays an important role in the tumor pathogenesis (27), being even responsible for the acquisition of therapeutic resistance (47–50). The stromal component consists of a variety of cells, including fibroblasts, pericytes, endothelial cells, adipose cells, but as fibroblast are the most common stromal component within tissues, most of the studies focus on them (51). There is evidence sustaining senescent fibroblasts to facilitate tumorigenesis of epithelial cancers by generating a tumor permissive microenvironment, promoting the growth of malignant cells of breast (3), skin (52, 53), prostate (54), colon (30), gastric (55) and oral cancers (10). In addition to fibroblasts, other stromal cells (including pericytes, adipocytes, lymphocytes, among others) have also been implicated in the generation of a tumor permissive microenvironment (56, 57).

The mechanisms underlying these pro-tumorigenic effects are not entirely known. As the organism ages, the fibroblast renewal rate decreases (58). Senescence of immune cells also reduces the clearance of other senescent cells (59). Therefore, senescent cells accumulate in tissues, resulting in an aged tumor microenvironment (TME) with abundancy of SASP factors (1). It is hypothesized then, that this would generate a switch towards a more immunosuppressive immune infiltrate (60, 61), establishing chronic inflammation, generating a microenvironment prone for cancer formation and progression. The factors that accumulate in an aged TME include proteins able to remodelate the extracellular matrix (ECM), such as MMPs, plasminogen activator inhibitors (PAI1 and PAI2), tissue-type plasminogen activator (tPA) and the secretion of pro-inflammatory molecules and growth factors, such as CXCL1, CXCL8, CXCL2, IL-6, IL-1α and colony stimulating factors (CSFs) (62).

The TME is not the only source of senescent cells. Tumor cells themselves can be induced to senesce by chemotherapeutic drugs (23) or by radiation therapy (63), which is known as therapy induced senescence (TIS). TIS does not only affect cancerous cells, but also stromal cells, and is considered an off-target effect of cancer therapy (64). In theory, TIS of cancerous cells should be a desirable outcome of cancer treatment because even when the treatment is not able to eliminate all malignant cells, the remaining living cells are not able to proliferate. Nevertheless, cancerous TIS cells remain metabolically active and together with other stromal TIS cells are a source of chronic inflammation through the SASP, facilitating drug resistance (65), tumor relapse (66) and distant metastasis (67).

## 4. Senescence in epithelial premalignancies

In human premalignancies, cellular senescence of the epithelial compartment is considered a tumor suppressor mechanism, as the acquisition of an indefinite replicative lifespan (one of the most important hallmarks of cancerous cells) depends on bypassing senescence via inactivation of p16^INK4A^ and p53 (44, 68), the main pathways responsible for senescence induction and maintenance (13). There is evidence that senescent cells accumulate *in vivo* in human premalignancies, such as colonic adenomas (69), cervical intraepithelial neoplasia (70), Bowen's disease (71), ductal carcinoma *in situ* (72), actinic keratosis (73) and oral potentially malignant disorder (OPMD) (74–76). OPMD encompass a heterogenous group of disorders characterized by an increased risk for developing cancer (77). The global prevalence of OPMD has been estimated in 4.47%. Oral leukoplakia (OL) and oral submucous fibrosis (OSMF) are the most common disorders (78), with a malignant transformation rate of 9.5% and 5.2% respectively (79).

A recent study that analyzed 50 OL samples with and without dysplasia found p16 positive keratinocytes in all 50 lesions, which were not related to HPV infection (76). Similarly, another study analyzed senescent markers in 86 OL with different degrees of dysplasia and found that both γH2AX and p53 proteins increase progressively according to the severity of dysplasia (75). There are also reports that senescent cells and DNA damage accumulate in higher numbers in many human premalignancies compared to their corresponding malignancies (80–83). In OL with dysplasia, senescent markers cyclin D1, maspin, Rb, and p16^INK4A^ have been found at higher levels compared to OSCC (74). All these data suggest that the epithelial senescence program prevents malignant transformation, which needs to be dismantled prior to cancer development.

**Figure 1:**
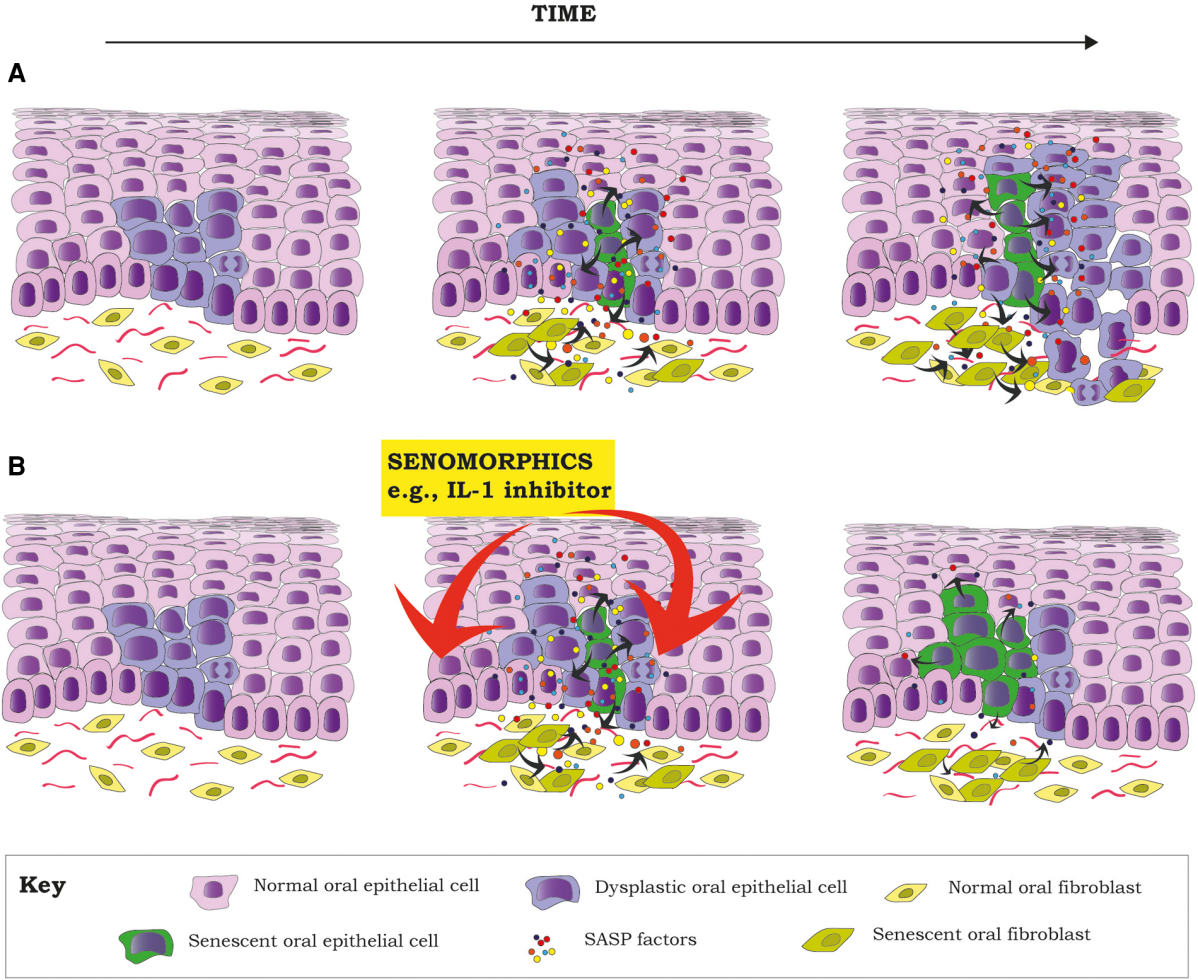
Senescent cells can create an environment for tumor promotion, by facilitating proliferation and the acquisition of a malignant phenotype of dysplastic cells (pre-malignant) through the secretion of different inflammatory factors as part of the SASP, a hallmark of senescent cells (**A**). Proposal of how the inhibition of different components of the SASP by senomorphics (e.g., IL-1 pathway) at the dysplasia state could decrease the SASP of senescent cells (both epithelial and stromal). This would potentially reduce the inflammatory component of the TME, reducing the chances of malignant transformation of already dysplastic cells (**B**). Red arrows mean inhibition.

Although senescence of the epithelial compartment has shown to be tumor suppressor, this does not mean that senescent epithelial cells are of no harm. In an OL with dysplasia, most, but not all dysplastic cells will senesce due to DNA damage response (DDR) due to oncogenic stress (oncogene induced senescence). But some cells will escape senescence and will become immortal, as has been shown in keratinocytes isolated from oral dysplasias (44). The acquisition of an immortal phenotype depends on p16 mutation or methylation, mutations or inactivation of p53 and reactivation of telomerase (44, 84). On the other side, dysplastic senescent keratinocytes will develop a SASP characterized by high levels of IL-6, IL-8 and IL-1α (cytokines with known oncogenic potential) (3, 85, 86) and other inflammatory and growth factors (87). These soluble factors can also induce paracrine senescence of surrounding normal keratinocytes, fibroblasts and other stromal cells (40), increasing the abundancy of SASP factors in the microenvironment. Thus, the “initiated” dysplastic keratinocytes that escaped senescence (immortal) will be exposed to an inflammatory pro tumorigenic microenvironment that can promote cancer development (46, 85) (Figure 1A). In fact, it has been recently shown that senescent mortal premalignant oral keratinocytes upregulate the expression of extracellular prostaglandins E1 and E2 (ePGE1 and 2), and that ePGE2, in conjunction with other SASP cytokines, are able to induce proliferation of immortal premalignant oral keratinocytes (88). If maintained over time, this could promote malignant transformation.

While in the epithelial compartment senescence is mainly considered a tumor suppressor mechanism (although as mentioned above it could also act a tumor promoter), senescence of surrounding stromal cells, such as fibroblasts, is considered tumor promoting (68). In skin, senescent fibroblasts can promote carcinogenesis of keratinocytes via factors present in the SASP (53). For example, pre-malignant breast epithelial cells can become invasive and suffer epithelial-to-mesenchymal transition (EMT) after stimulation by SASP factors from surrounding senescent fibroblasts via a paracrine mechanism dependent of IL-6 and IL-8 (3). Pre-malignant non-tumorigenic human embryonic kidney cells, in the presence of SASP factors from senescent fibroblast, can also become tumorigenic with stem-like properties (89). Senescent prostatic fibroblasts are able to induce proliferation of prostate epithelial cells through a paracrine mechanism including hepatocyte growth factor (HGF), fibroblast growth factor 7 (FGF7) and amphiregulin (AREG) (54). GDF15, an essential factor from the SASP from senescent colonic fibroblasts, can promote malignant features (increased proliferation, migration, and invasion) in colon adenoma cell lines (30).

Whilst there is substantial evidence supporting a cancer promoting role of senescent fibroblast in different premalignancies, there is scarce information about their role in OPMD, being most studies performed in OSMF, in which cellular senescence has been proposed to have an essential role in the malignant transformation (90). OSMF is a progressive fibrotic disease caused by the chewing of areca nut characterized by a gradual reduction in the jaw opening (91) and an increased risk for oral cancer development (92). In the initial phases, reactive oxygen species (ROS) induce senescence of epithelial stem cells leading to epithelial atrophy (90, 93). This is followed by senescence of endothelial cells and fibroblasts (90), which reduces tissue vascularity generating hypoxia and fibrosis. Alkaloids and flavonoids from areca nut also stimulate fibroblasts to produce more collagen. This leads to more collagen deposition affecting deeper tissues as the disease progresses (46). As fibrosis increases, the epithelium atrophies due to senescence, and senescent fibroblasts accumulate in the stroma (90), which precedes the development of epithelial dysplasia (51). For the malignant transformation to happen, the dysplastic senescent epithelial cells must escape senescence, likely due to upregulation of LOLXL2 ad mucin 4 (94, 95). This will result in immortal dysplastic epithelial cells exposed to upregulated SASP factors secreted by senescent fibroblast, such as IL-1, IL-6, IL-8 and GRO-α/CXCL-1, which will increase ROS production, generating DSB, favoring cancer development (96).

## 5. Senescence and OSCC

In OSCC, most of the epithelial cancerous cells have escape senescence to become malignant through TP53 and p16^INKA4^ mutations, although a subset of cancerous cells has been reported to retain functional Tp53 or p16^INKA4^ (97, 98). Nevertheless, those mortal cancerous cells are expected to acquire further mutations to reach immortality or will disappear due to natural selection. Therefore, senescence of malignant keratinocytes is not naturally expected in OSCCs or should not have a significant effect in tumor behavior (unless the tumor is irradiated or treated with chemotherapeutic drugs). But as mentioned earlier, senescence of stromal components of the tumor can have deleterious effects. The most studied stromal cells in OSCCs are cancer-associated fibroblasts (CAF).

CAF are a poorly characterized heterogenous cell population with different subtypes, including activated myofibroblastic CAF (myCAF) and senescent fibroblasts (99, 100). There is substantial evidence that the accumulation of myCAF is associated with a poor prognosis in OSCC (83, 101), as myCAF support OSCC progression, tumor growth and invasion (99). Senescent oral fibroblasts have also been reported to have pro-tumorigenic effects in OSCC (10, 11, 100). They share an overlapping gene expression profile with myCAF (100), suggesting that these CAF phenotypes are closely related. Senescent fibroblasts are found in the stroma of OSCC in different quantities (74, 100) and have been reported to be in higher numbers than in normal oral mucosa and dysplastic oral lesions (10). Interestingly, the injection of small number of senescent cells into mice caused persistent physical dysfunction with shorter health and life span, indicating that the number of senescent cells is not important (102). Furthermore, the injection of senescent cells spread the senescence to host tissues (102), probably in a paracrine manner through components of the SASP (40, 103).

The accumulation of senescent fibroblast in OSCC is an early event unlikely to be due to replicative exhaustion, as some same age patients with OSCC have shown no senescent fibroblasts (10). Additionally, senescent fibroblasts also accumulate in OPMD in younger patients by a telomere-independent mechanism (93). Hassona et al. (2013) suggest that in OSCC, fibroblast senescence is caused by oxidative DNA damage. This would be induced by ROS produced by oral keratinocytes and fibroblasts from genetically unstable OSCC (developed from premalignancies with loss of *TP53* and P16*INK4A*), but not genetically stable OSCC (developed from premalignancies with functional p53), in a TGF-β dependent manner (10). This is supported by the fact that keratinocytes from genetically unstable OSCC produce higher amounts of ROS and are deficient in antioxidant defenses, suggesting that p53 functionality is important in the regulation of ROS production (10). Recently, it has been also proposed that impairment of the autophagy process might be also responsible for the development of the senescent and myofibroblastic phenotypes in CAF (104).

Regardless the mechanism underlying the induction of fibroblast senescence, the SASP from senescent oral fibroblast has been shown to have pro-tumorigenic effects on malignant oral keratinocytes (10, 11, 68, 100). SASP factors MMP-2 (11), ROS, TGF-β (10), PGE2 and the miR-335/COX-2/PTEN axis (105) have been shown to induce dis-cohesion, EMT, migration and invasion of oral malignant keratinocytes (10, 11, 68, 100, 105). A recent paper also showed that both, early senescence and NF-kB-dependent SASP cytokines secreted by senescent OSCC cells induced to senesce by radiotherapy, are critical for radioresistance in OSCC *in vivo* (12).

## 6. Discussion

Cellular aging is a relatively new research field that has developed during the last two decades and has gained increasing interest due to its relationship with organism aging and age-related disorders. Although initially cellular senescence was considered only as a potent anti-tumor mechanism, nowadays is also recognized as a potent tumor promoter. Consistent with this idea, senescent cells have been recently considered as an emerging hallmark of cancer with enabling characteristics (27) despite their well-known anti-tumorigenic functions.

Senescent cells might not only influence cancer behavior but also affect tolerance to cancer treatment, as the expression of a senescence marker in circulating T-cells before chemotherapy was associated with increased risk of chemotherapy-induced fatigue in humans. In addition, *in vivo* models in mice showed that eliminating TIS cells reduced short-and long-term effects of chemotherapeutic drugs, including cardiac dysfunction, physical activity, strength, bone marrow suppression and cancer recurrence (23).

This interest has led to the development of new drugs or to the finding of new uses for old drugs to specifically eliminate senescent cells or to target their SASP (senolytics and senomorphics respectively). This field, also known as senotherapeutics, has also emerged as new complimentary treatment alternative for some cancers.

Senolytic drugs consist usually of small molecule agents that target specifically anti-apoptotic pathways that are overexpressed in senescent cells as a pro-survival mechanism, but not in proliferating nor quiescent cells (13). There are different drugs targeting different components of the antiapoptotic pathways including: Navitoclax and ABT-737, both targeting the BCL-2 pathway (106, 107), Dasatinib, a tyrosine kinase inhibitor (108), AT-406, a regulator of anti-apoptotic proteins c-IAP2 and XIAP (109). These drugs have eliminated senescent cells in *in vitro* and in *in vivo* models improving clinical outcomes. For example, Navitoclax, has shown to eliminate ovarian and breast cancer TIS cells after PARP inhibitor therapy (110) and to induce tumor regression and improve therapeutic outcomes following conventional chemotherapy in mouse models (111). It has also shown to improve radiation-induced salivary gland hypofunction in irradiated mice by eliminating salivary gland senescent stem cells (112). Dasatinib, in combination with quercetin, reduces the population of senescent cells in mice attenuating adipose tissue inflammation, improving systemic metabolic function (108). The same combination of drugs has also been tested in clinical trials for the treatment of pulmonary fibrosis improving patient's physical function (9).

Senomorphics or SASP inhibitors are drugs aimed to decrease the pro-tumorigenic inflammatory component of the SASP, with the advantage of conserving pro-immunogenic functions of senescent cells, such as immunosurveillance. The main disadvantage of senomorphics over senolytics is that, as these drugs do not eliminate senescent cells, they might require long-term administration as their effect vanishes upon discontinuation (21). NF-*κ*B is probably the most important signaling pathway mediating the inflammatory components of the SASP. Thus, most studies have explored the use of drugs targeting this transcription pathway or NF-*κ*B-mediated cytokines, including metformin (113, 114), aventhramice C (115), Anakinra (IL-1 inhibitor) (116), Canakinumab (anti-IL-1β antibody) (117) or Simvastatin (IL-6 and IL-8 inhibitor) (118). Furthermore, the use of agents targeting other pathways such as Rho-kinase (Y27632) (41, 119), cGAS-STING (RU.521 (85), mTOR (Rapamycin) (31, 33), among many others, have also been assessed.

Inflammation seems to be of importance in OSCC development and progression (120). In solid tumors, the presence of senescent cells is one of the most important sources of inflammation. Therefore, targeting senescent cells to reduce inflammation seems a promising approach to find new treatment alternatives to improve treatment success. The increased expression of NF-*κ*B signaling pathway (121, 122) and NF-*κ*B-mediated cytokines (120), such as IL-1 in OPMD and OSCC, are reported to have impact in clinical outcomes (120, 123). IL-1 is considered a master regulator of the SASP and for the spread of paracrine senescence (40), and there is evidence from *in vivo* animal studies that IL-1 induces malignant transformation of oral precursor lesions and OSCC aggressiveness (124). Therefore, targeting senescent cells to reduce the overexpression of IL-1 with senolytics or senomorphics, in addition to surgical or chemotherapeutic treatment, could represent a novel treatment alternative for OPMDS and OSCC (Figure 1B). Although there are different clinical trials testing senolytics as single agents or in combination with other chemotherapeutic drugs for the treatment of different cancers, including lymphomas, melanoma, leukemia, lung, ovarian and prostate cancers (source: clinicaltrials.gov), more *in vitro* and *in vivo* animal studies are needed to support the use of senotherapeutics for OSCC treatment.
